# Expression patterns and prognostic implications of tumor-infiltrating lymphocytes dynamics in early breast cancer patients receiving neoadjuvant therapy: A systematic review and meta-analysis

**DOI:** 10.3389/fonc.2022.999843

**Published:** 2022-11-30

**Authors:** Yajing Zhu, Evangelos Tzoras, Alexios Matikas, Jonas Bergh, Antonios Valachis, Ioannis Zerdes, Theodoros Foukakis

**Affiliations:** ^1^ Department of Oncology-Pathology, Karolinska Institutet, Stockholm, Sweden; ^2^ Breast Center, Theme Cancer, Karolinska University Hospital, Stockholm, Sweden; ^3^ Department of Oncology, Faculty of Medicine and Health, Örebro University, Örebro, Sweden

**Keywords:** tumor-infiltrating lymphocytes (TILs), TILs dynamics, breast cancer, biomarker, neoadjuvant treatment, prognosis

## Abstract

**Purpose:**

High levels of tumor-infiltrating lymphocytes (TILs) are associated with better outcomes in early breast cancer and higher pathological response rates to neoadjuvant chemotherapy especially in the triple-negative (TNBC) and HER2+ subtypes. However, the dynamic changes in TILs levels after neoadjuvant treatment (NAT) are less studied. This systematic review and meta-analysis aimed to investigate the patterns and role of TILs dynamics change in early breast cancer patients receiving NAT.

**Methods:**

Medline, Embase, Web of Science Core Collection and PubMed Central databases were searched for eligible studies. Data were extracted independently by two researchers and discordances were resolved by a third. Pooled TILs rates pre- & post-treatment (overall and per subtype), pooled rates of ΔTILs and direction of change after NAT as well as correlation of ΔTILs with survival outcomes were generated in the outcome analysis.

**Results:**

Of 2116 identified entries, 34 studies fulfilled the criteria and provided adequate data for the outcomes of interest. A decreased level of TILs was observed after NAT in paired samples across all subtypes. The effect of NAT on TILs was most prominent in TNBC subtype with a substantial change, either increase or decrease, in 79.3% (95% CI 61.7-92.6%) of the patients as well as in HER2+ disease (14.4% increased vs 46.2% decreased). An increase in ΔTILs in TNBC was associated with better disease-free/relapse-free survival in pooled analysis (univariate HR = 0.59, 95% CI: 0.37–0.95, p = 0.03).

**Conclusion:**

This meta-analysis illustrates the TILs dynamics during NAT for breast cancer and indicates prognostic implications of ΔTILs in TNBC. The potential clinical utility of the longitudinal assessment of TILs during neoadjuvant therapy warrants further validation.

## Introduction

Breast cancer (BC) is the most frequently diagnosed cancer and the leading cause of cancer-related death among women worldwide (1). While neoadjuvant and adjuvant chemotherapy clearly improve patient outcomes, clinical-pathologic factors and available gene signatures failed to demonstrate validated predictive value for chemotherapy benefit (2, 3). We have previously shown that immune-related gene expression is both prognostic and predictive for chemotherapy benefit in early and advanced BC (4–8). However, using immune gene signatures in the clinical routine is complex due to the lack of standardized and prospectively validated methods and the lack of estimations on potential health impact and costs.

A simple-to-use and widely available immune biomarker is the number of tumor-infiltrating lymphocytes (TILs) on hematoxylin eosin (H&E) stained tissue sections. It has been previously described that high TIL infiltration at diagnosis was strongly associated with a better response to neoadjuvant chemotherapy (NACT) (9). Several subsequent studies reported the positive predictive and prognostic value of primary TILs both in the neoadjuvant and adjuvant setting, especially for TNBC and HER2+ tumors (10–12). In order to mitigate interobserver variability, the International Immuno-Oncology Biomarker Working Group has established guidelines for the standardized evaluation of TILs (13, 14). The latest edition of the WHO classification of tumors has introduced TILs as an important prognostic marker (15) whereas some currently on-going prospective trials include TILs as a pre-specified stratification factor in TNBC and HER2+ patients receiving neoadjuvant treatment (16, 17).

The current evidence on TILs is mainly based on a cross sectional evaluation where the level of TILs is assessed once, usually before any systemic treatment is administered. However, a dynamic, longitudinal evaluation of immunological markers may give us better understanding of the mechanisms that govern the host response to tumor and be a potential source of clinically useful biomarkers. Some studies have investigated both pre-treatment and post-treatment TILs in paired tissues during neoadjuvant chemotherapy and the association of TILs change with prognosis, with conflicting results.

The aim of the present meta-analysis was to gather the current evidence on TIL dynamics following neoadjuvant therapy and investigate the magnitude and direction of TILs changes as well as their correlation with therapy prediction and survival outcomes.

## Methods

### Search strategy and study selection

A comprehensive literature search was conducted by the Karolinska Institutet University Library in May 2020 and updated in September 2021. The following four databases were searched: Medline (Ovid), Embase (embase.com), Web of Science Core Collection and PubMed Central. The MeSH (Medical Subject Headings) terms used were: Breast Neoplasms, Lymphocytes, Tumor-Infiltrating, CD3 Complex, Neoadjuvant Therapy, Chemotherapy, Adjuvant. The MeSH terms for searching Medline (Ovid) were adapted in accordance with corresponding vocabulary in Embase. Databases were searched from inception. The detailed search strategies are provided in **Supplementary Data**.

Studies included in our meta-analysis were restricted to English and fulfilled at least one of the following criteria (1): Stromal TILs evaluated in paired human breast cancer tumor samples before and after neoadjuvant chemotherapy, targeted and/or endocrine therapy (2); TILs evaluated in paired human breast cancer tumor samples before and during neoadjuvant chemotherapy targeted and/or endocrine therapy; (3) relationship between ΔTILs levels and short-/long-term prognosis in non-pCR and pCR cases; (4) relationship between ΔTILs levels and pCR (for pCR patient cases, TILs were measured on tissue scar or tumor bed area). TILs could have been reported as continuous or categorical variables and assessed on H&E slides, regardless of methods used, including manual evaluation or digital image analysis. If both intra-tumoral and stromal TILs were evaluated, only stromal TILs information was included for analysis. ΔTILs is defined as change in median/mean lymphocyte density between pre- and post-treatment samples; ΔTILs was either reported in the articles or calculated manually in articles with relevant data.

Studies were excluded if they met at least one of the following criteria: (1) reviews, commentaries, editorials, conference abstracts, protocols, case reports, qualitative research, or letters; (2) duplicate publications/entries; (3) full text not published in English. Study selection was performed independently by two investigators (Y. Zhu and E. Tzoras) and consensus was reached in all eligible studies.

### Data extraction

Two investigators (Y. Zhu and E. Tzoras) independently extracted the data to a predefined form and a third investigator (I. Zerdes) resolved any discrepancies. The concordance rate between the two investigators was 86%. Data collected from each study included: first author’s last name, journal name, year of publication, country where the study was conducted, type of study (retrospective/prospective), enrolment dates, number of evaluable patients before NAT, number of evaluable patients after NAT, number of patients with matched- paired samples, tissue used for analysis (tissue microarrays, whole-tissue sections), method used for analysis, threshold for positivity/high expression of stromal TILs, median/mean TILs level before NAT, median/mean TILs level after NAT, ΔTILs mean change, absolute number of patients with increased/decreased/unchanged TILs, % TILs in matched pre- and post-NAT samples and change-ΔTILs if reported, characteristics of study cohort, follow-up time; outcomes (pCR and time-to-event endpoints) within all patients and whenever possible within different breast cancer subtypes including both univariate and multivariate results.

### Quality assessment

Two investigators (Y. Zhu and E. Tzoras) independently assessed each eligible study for methodological quality using the 20-item REMARK checklist (18) and the discrepancies were resolved by a third investigator (I. Zerdes). The REMARK checklist consists of 20 items to report in tumor marker prognostic studies evaluating several aspects of study quality from scientific rationale and result interpretation to study design and methodology used. Each of the 20 items listed in REMARK was scored with 0 (not defined or inadequate defined or not applicable), 1 (incomplete or unclear defined), or 2 (clearly defined) for each eligible study, with a maximum score of 40. No studies were excluded based on quality control.

### Statistical analysis

High and low TILs were defined according to cut-offs described in each article for articles reported TILs as a categorical variable.

For analyses of pooled expression of TILs in matched breast cancer patients in studies presented TILs as categorical variable, a random-effects model was used to calculate the pooled high-level TILs and corresponding 95% confidence interval (CI) pre- vs. post-treatment for different breast cancer subtypes (HER2-positive, TNBC, luminal, not specified [contain studies recruit all BC patients without limitation of molecular subtype]). An overall effect estimate was thereafter calculated using Odds Ratio (OR) with 95% CI through the DerSimonian and Laird method (19).

For pooled analyses of difference in TILs expression pre- vs. post-treatment when TILs were presented as continuous variables, standardized mean differences (SMD) with 95% CI were calculated for each study and then pooled to present a measure of the effect size of the difference in TILs in pre- and post- treatment groups.

For the comparisons of time-to-event variables based on the direction of TILs changes, a meta-analysis was performed first by transforming the Hazard ratios (HRs) and their errors into their log counterparts, and then using the inverse variance method for transforming back into the HR scale. If adequate data from time-to-event variables were unavailable for direct extraction from the primary studies, data were extracted according to the method described by Tierney et al. (20). A pooled analysis was performed only if at least three primary studies presented adequate data for analyses.

The presence of statistical heterogeneity among the studies was addressed by using the Q statistics, and the magnitude of heterogeneity by using the I² statistic. A p-value < 0.10 or a I² value of greater than 50% was considered as substantial statistical heterogeneity. Considering the substantial clinical heterogeneity among eligible studies, all meta-analyses except the one with time-to-event variable as outcome of interest were performed using random-effects models. The presence of publication bias was evaluated qualitatively using a funnel plot.

All reported p values are two sided. Analyses were conducted on RevMan 5.3 (Review Manager, Version 5.3; The Cochrane Collaboration, 2014) and on StatsDirect (StatsDirect Ltd. StatsDirect statistical software. http://www.statsdirect.com. England: StatsDirect Ltd. 2013).

## Results

### Study characteristics

**Figure 1:**
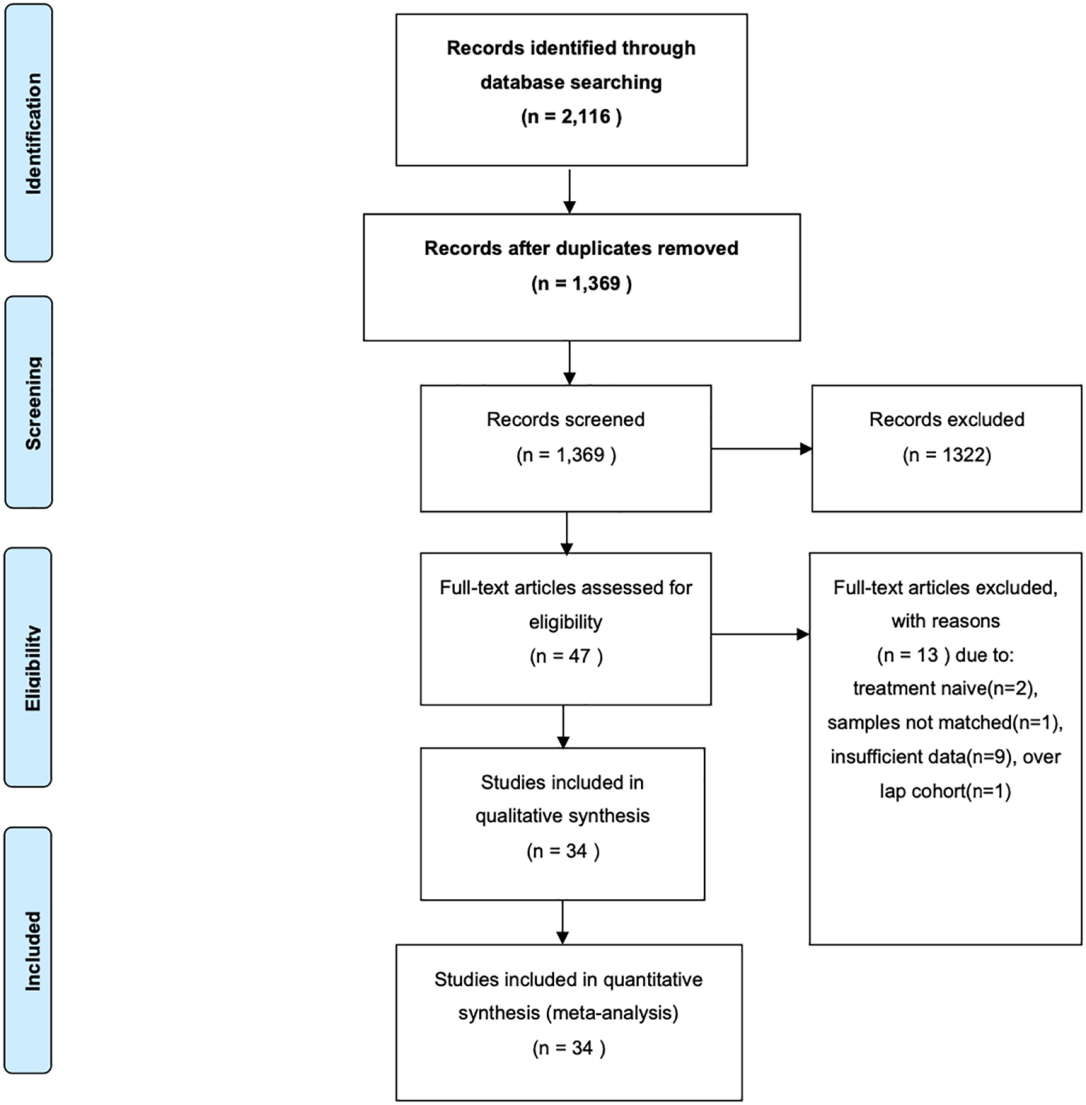
Flow Diagram of search and study selection in this meta-analysis.

The flow diagram of study selection for the study-level meta-analysis is shown in **Figure 1**. The initial search identified 2,116 entries, or 1,369 entries following deduplication. Through exclusion by reading the title and/or abstract, 47 possibly eligible studies were retrieved as full text; In total, 34 studies fulfilled the inclusion criteria and were included for various meta-analytic questions. All 34 studies were included for pooled pre- and post-TILs change direction analysis (separate analysis for TILs as categorical variable [14 studies] and continuous variable [21 studies]); 26 studies reporting matched paired breast cancer patients were included for pooled rates of ΔTILs. 4 studies reported survival information and were included for pooled HR analysis of ΔTILs and prognosis association. The detailed characteristics of eligible studies are presented in **Table 1**.

**Table 1:** Characteristics of studies included in the meta-analysis.

Author [reference]	Year	Country	Variable type	No. of matched paired pts	BC subtype	NAT regimen	TILs Cutoffs	Median follow-up	QC
Honkoop (21)	1996	Netherlands	Categorical	11	All	AC	Absent: – Present:+ Abundant: ++	NA	15
Abdel-Fatah (22)	2014	UK	Categorical	196	All	FEC/FAC, FEC→T, T-FEC, EC+T, EC+GT, AC-T, AC, T	Predominant: >60% Focal: 10-60% Minimal: <10%	51 (6–170) mons	24
Dieci (23)	2014	Italy	Categorical	19	TNBC	A/T	LPBC: >60% Low: <60%	6.3 yrs	36
Ali (24)	2016	UK	Continuous	557	All	G ± E/C→T	NA	NA	35
Castaneda (25)	2016	Peru	Categorical& continuous	89	TNBC	A/T	High: >50%	37.5 mons	22
Criscitello (26)	2016	Italy	Continuous	29	ER-/HER2-	AC+T/AX→FEC/T→FAC	NA	NA	27
Dieci (27)	2016	Italy	Categorical& continuous	57	HER2+	T→FEC + Tr/L/tr+L	LPBC: >60%	NA	32
Hida (28)	2016	Japan	Categorical	58	TNBC, HER2+	AT ± Tr or EnT	Low: <10% IM: 10-50% High: >50%	TNBC 23 mons (IQR 12.5 - 37) HER2+ 34 mons (IQR 23.8 - 48.3)	32
Kaewkangsadan (29)	2016	UK	Categorical	16	All	AC→T ± X	High: > 60%	NA	32
Park (30)	2016	Korea	Categorical& continuous	24	ER+/HER2+	L+LET	High: >20%	28.5 mons	29
Goto (31)	2017	Japan	Categorical	129	All	FEC→T ± Tr	High: >10%	NA	28
Hamy (32)	2017	France	Categorical& continuous	175	HER2+	A-based/AT- based ± Tr	Low:< 10% IM: 10-60% LPBC: >60%	38.8 mons (range 5.5-91.7 mons)	35
Pelekanou (33)	2017	USA	Continuous	43	All	AC→T	Negative: <1% Positive: >1% LPBC: >50%	NA	26
Force (34)	2018	USA	Categorical& continuous	30	HER2+	T+Pt+Tr ± P	LPBC: >50%	NA	18
Hwang (35)	2018	Korea	Categorical	204	All	AT ± tras/pertu or ET	LPBC: >50%	60.1 mons	35
Pelekanou (36)	2018	USA	Categorical& continuous	59	HER2-	T+AC ± Bev	Negative: <1% Positive: >1% LPBC: >50%	3 yrs	30
Watanabe (37)	2018	Japan	Categorical	139	All	AT ± anti-HER2 therapy	Low: <10% IM:10-50% LPBC: >50%	pre pts: 24.5 m (range 13-45.6 m) post pts: 26.1 m (range 13.5 - 48.8 mons)	28
Di Cosimo (38)	2019	Italy	Continuous	11	TNBC	AT	NA	70 mons (50-81 mons)	18
Hamy (39)	2019	France	Categorical& continuous	718	All	AT ± Tr/EnT	High: >60%	NA	37
Kurozumi (40)	2019	Japan	Categorical	45	HER2+	AT+Tr	Low: 0-10% IM: 10-40% High: 40-90%	NA	29
Liu (41)	2019	China	Continuous	19	All	EC→T/TEC+Tr	NA	40 mons (range 34-47mons)	28
Luen (42)	2019	Australia	Categorical& continuous	163	TNBC	AT	High: >20%	6 yrs	36
Ochi (43)	2019	Japan	Categorical	130	TNBC and HER2+	AT ± Tr	Low:0-9% IM:10-49% LPBC:> 50%	98 mons (range 2 - 120 mons)	35
Tokés (44)	2019	Hungary	Categorical& continuous	120	All	A-based/Pt-based/C-based regimens	Positive: >1% LPBC: >50% Negative <1%	31.1 mons (range 2.6-120.4 mons)	36
Wang (45)	2019	China	Categorical& continuous	75	All	ACT ± Tr	Positive: > 1% Negative: <1%	23.2 mons range (6.1-64.5 mons)	32
Abdelrahman (46)	2020	Egypt	Categorical	30	TNBC	ChT	High: > 50%	NA	28
Axelrod (47)	2020	USA	Categorical& continuous	83	All	ChT ± Tr	High: > 30%	NA	26
Cambedel (48)	2020	France	Continuous	31	TNBC	AC ± T	NA	49 mons	35
Grandal (49)	2020	France	Continuous	192	All	AT	NA	90.4 mons (0.2 -187 mons)	26
Kim (50)	2020	Japan	Categorical& continuous	43	All	FEC/FEC+T/EC+T ± Tr	Positive: >5%	NA	25
Lee (51)	2020	Korea	Continuous	104	TNBC	AT	NA	72.3 mons	28
Park (52)	2020	Korea	Continuous	26	All	AC→T ± Tr	NA	NA	29
Saradin (53)	2021	France	Categorical& continuous	66	TNBC	FEC/EC+T	High: > 10% or > 30%	35.4 mons [95% CI 26.5–44.4]	21
Grandal (54)	2021	France	Continuous	87	TNBC	AT	NA	80 mons	28

LPBC, Lymphocyte predominant breast cancer; IM, intermediate; A, anthracycline; C: cyclophosphamide; T, taxanes; Fu, fluorouracil; X, capecitabine; G, gemcitabine; Pt, platinum; AI, aromatase inhibitor; L, lapatinib; LET, letrozole; Tr, trastuzumab; P, pertuzumab; Bev: bevacizumab; ChT, chemotherapy; EnT, endocrine therapy; Mons, months Yrs, years; NA, not available.

### Quality of eligible studies, between-study heterogeneity and assessment of publication bias

All eligible studies for the meta-analysis were retrospective. The median number of study quality score was 29 (range: 15-37) out of a maximum score of 40. Substantial between-study heterogeneity was noted among eligible studies regarding the breast cancer subtypes, treatment regimens used, variable types used to report TILs level, and the follow-up period. The risk of publication bias for the pooled estimates was visually assessed by funnel plots. With reservation due the low number of primary studies in some pooled estimates, no evidence of asymmetry was observed in funnel plots implying a lower risk for publication bias (**Supplementary Figure 1**).

### Pooled TILs expression before and after neoadjuvant treatment

The number of studies and patient cases with available information on TILs as categorical variable across BC subtypes, as well as the pooled rates of high-level TILs are presented in **Table 2**. The proportion of cases classified as high-level TILs decreased post-treatment across BC subtypes, although no pooled analysis was possible for the Luminal subtype due to the low number of studies (Not specified: pooled OR [95% CI] = 1.60 [95% CI: 1.12-2.30]; HER2-positive: pooled OR [95% CI] =1.88 [0.87-4.08); TNBC: pooled OR [95% CI] =1.05 [0.41-2.68]. Difference in pooled rates of TILs pre- vs. post-treatment was statistically significant for the “not specified” subgroup.

**Table 2:** Pooled expression of TILs pre- vs. post-treatment in matched breast cancer patients in studies presented TILs as categorical variable.

Breast cancer subtype	N studies (n paired cases)	pooled high-level TILs (95% CI)		Pooled Odds ratio (95% CI)	I^2^
		*Pre-treatment*	*Post-treatment*		
Not specified	5 (431)	27.5 (16.3-40.4)	17.0 (7.4-29.4)	1.60 (1.12-2.30)	40.5
Luminal	2 (184)	NC	NC	NC	NC
HER2-positive	3 (93)	20.6 (13.3-29.0)	12.2 (4.1-23.8)	1.88 (0.87-4.08)	0
TNBC	4 (139)	21.4 (15.1-28.5)	15.7 (3.7-34.3)	1.05 (0.41-2.68)	42.7

CI, confidence intervals; NC, not calculated.

**Figure 2:**
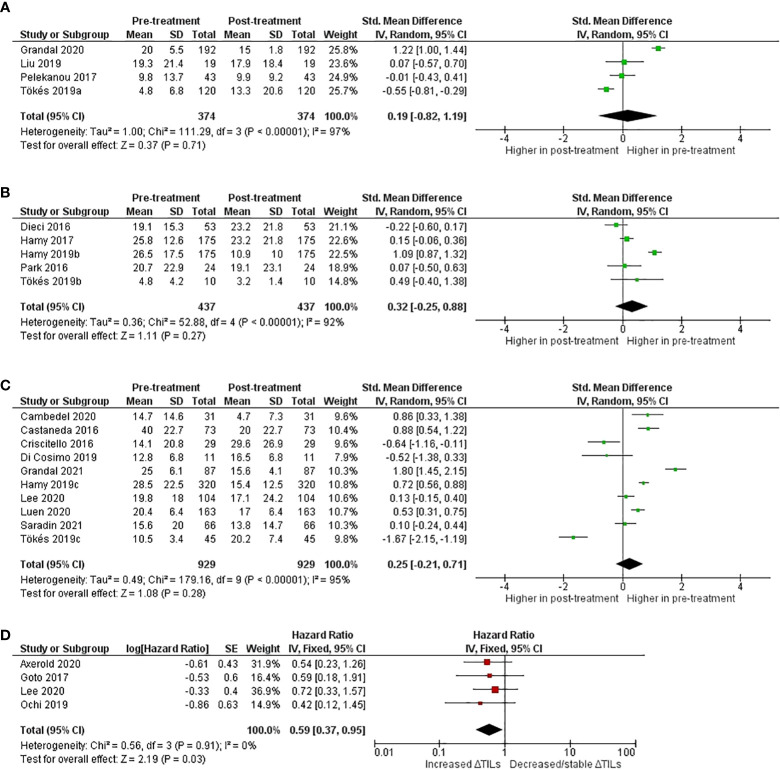
Forest-plots on Standardized mean difference (SMD) of Tumor-Infiltrating Lymphocytes (TILs) pre- and post-treatment per breast cancer subtype **(A)** not specified **(B)** HER2-positive **(C)** Triple-negative breast cancer [TNBC] and **(D)** Disease-free survival [DFS]/Recurrence-free survival [RFS] according to TILs change in TNBC subtype.

Furthermore, twenty-one studies reported TILs as continuous variable. Number of studies, cases, pooled standardized mean difference (SMD) and I^2^ in four subgroups were summarized in **Supplementary Table 1**. Positive SMD values were seen in the HER2+, TNBC and not specific subgroups while no pooled analysis was done for the Luminal subtype due to only two studies have available data. Forest plots on pooled SMD pre- and post-treatment in studies with each BC subtype are shown in **Figures 2A–C**. Although the magnitude of pooled effect sizes is not statistically significant, numerically higher TILs expression at pre-treatment compared to post-treatment was seen in all three subgroups.

### Pooled ΔTILs rates after neoadjuvant therapy in early BC patients

Studies evaluating ΔTILs in matched pre-treatment and surgical samples from the same patients were also included in this analysis from all/unspecified (10 studies; n=1758), luminal (1 study; n=106), HER2-positive (6 studies; n=414), TNBC (9 studies; n=483) subtypes. Change in TILs following neoadjuvant therapy was bi-directional mainly in TNBC cases, whereas TILs mostly decreased post-therapy in the mixed and HER2-positive populations (**Table 3**).

**Table 3:** Pooled rates of ΔTILs before and after NAT in matched-paired tissue samples in early breast cancer patients.

Breast cancer subtype	N studies (n paired cases)	pooled rates of ΔTILs (95% CI)		
		Increased TILs	Decreased TILs	Changed at any direction
Not specified	10 (1758)	30.3 (21.9-39.5)	49.4 (36.5-62.4)	85.7 (68.6-96.7)
Luminal	1 (106)	NC	NC	NC
HER2-positive	6 (414)	14.4 (8.9-21.0)	46.2 (20.0-73.7)	66.2 (34.3-91.5)
TNBC	9 (483)	41.6 (28.4-55.5)	37.1 (26.8-47.9)	79.3 (61.7-92.6)

CI, confidence intervals; NC, not calculated.

### Prognostic implications of TILs change after neoadjuvant therapy

Pooled HRs from univariate analyses for disease-free survival (DFS) or Recurrence-free survival (RFS) for TNBC patients from 4 eligible studies are presented in **Figure 2D**. For this outcome, we considered different definitions of DFS or RFS as similar and analyzed within the same meta-analysis. Two studies defined RFS as the time from the date of primary surgery until the date of disease recurrence (31, 47), one study defined RFS as time from diagnosis to locoregional recurrence, distant metastasis, or death from any cause (43) whereas no clear definition was described in one study (51). Increased ΔTILs was associated with better DFS/RFS with a pooled HR of 0.59 (95% CI: 0.37 – 0.95, p = 0.03). Because of data paucity, meta-analysis in other BC subtypes, or pooled HR from multivariate analyses, could not be performed.

### Studies assessing on-treatment TILs and correlation with pCR

Eight prospective studies retrospectively assessed TILs before and during neoadjuvant treatment were identified from the systematic literature review. Within these 8 studies, 5 included HER2-positive breast cancer patients that received HER2-targeted therapy with or without chemotherapy (55–59), 1 study included TNBC patients who received combination of immunotherapy and chemotherapy (60), 1 study included hormone receptor positive breast cancer patients received chemotherapy combined with anti-angiogenesis therapy (5) and 1 study included non-specific patients received chemotherapy with or without HER2-trageted therapy (52) (**Table 4**). On-treatment TILs counts uniformly increased compared with baseline status. With the exception of one study (57), increased TILs between pre- and on-treatment biopsies were positively associated with pCR status. Pooled analyses were not possible due to inadequate number of studies per breast cancer subtype and heterogeneity among the eligible studies.

**Table 4:** Studies assessed the expression of on-treatment TILs in matched-paired early breast cancer patients receiving neoadjuvant therapy.

Author[reference]	Year	Country	No. of Matched paired patients	BC sub type	Variable	Cut-off or LPBC	ΔTILs Direction(On–Pre)	Mean ΔTILs(On–Pre)	Increase TILs-response	NAT	QC
Loibl (55)	2017	Germany	36	HER2-positive	Continuous	NA	Increase	B. +11.87% Placebo +12.75%	pCR	B+Tr→T vs Placebo+Tr→T	27
Nuciforo (56)	2017	Spain	131	HER2-positive	Continuous	50%	Increase	6.93%	pCR	Tr+L ± EnT	32
Matikas (5)	2018	Sweden	41	Luminal	Continuous	50%	NA	NA	NA	AT ± Bev	25
Loibl (60)	2019	Germany	81	TNBC	Continuous	NA	Increase	D. +5.8% Placebo +4.1%	pCR	D→D+Nab-p vs D→placebo+Nab-p	36
Park (52)	2020	S. Korea	98	All	Continuous	NA	Increase	NA	pCR	AC→T ± Tr	36
Hurvitz (59)	2020	USA	55	HER2-positive	Continuous	NA	Increase	+2.8%	NA	Tr/L/Tr+L→Tr/L/Tr+L + T + Pt	24
Eustace (57)	2021	Ireland	16	HER2-positive	Continuous	NA	Increase for RD	NA	RD	Tr+T+Pt/Tr+L+T+Pt	29
Griguolo (58)	2021	Italy	131	HER2-positive	Continuous	NA	Increase	NA	NA	Tr+L ± EnT	33

Tr, trasuzumab; L, lapatinib; T, taxane: Nab-p, nab-paclitaxel; Pt, platinum; A, anthracycline; C, cyclophosphamide; D, durvalumab; B, Buparlisib; EnT, endocrine treatment; Bev: bevacizumab; Tr, trastuzumab; L, lapatinib; LPBC, lymphocyte predominant breast cancer; pCR, pathologic complete response: RD, residual disease; NA, not available.

## Discussion

This meta-analysis summarizes the current evidence on pooled TILs levels in matched paired tissues before and after NAT in breast cancer patients and presents data related to dynamic changes of TILs during NAT. Higher TILs expression at pre-treatment compared to post-treatment was seen across all BC subgroups with consistent results in studies reported TILs as categorical or continuous variable types, with a more distinct decreasing trend seen in HER2-positive subgroup. By pooling data from around 450 TNBC patients, we also reported a positive correlation of increased ΔTILs with improved survival, though confounding bias cannot be excluded.

Our study provides some interesting insights on how TILs could be potentially used to better optimize neoadjuvant treatment mainly for TNBC and HER2+ patients.

First, a trend towards decreased TILs after NAT was observed in all pooled analyses irrespectively breast cancer subtype. Although this trend is small and not statistically significant in most of the analyses, the consistency of the decreased trend across all breast cancer subtypes implies a potential true effect. Since all included studies except for one (30) used neoadjuvant regimens containing at least one chemotherapeutic agent, the decreased TILs seen in our findings may be driven by the treatment effect of cytotoxic chemotherapy, which is generally considered to be immunosuppressive (61). Considering the diversity of chemotherapeutic agents used in eligible studies, no firm conclusion can be made on how different chemotherapeutic agents could affect immune response. In fact, some recent studies suggest that different chemotherapeutic agents might have distinct effect on immune cell surface marker expression (62) whereas some third-generation cytotoxic drugs such as pemetrexed can potentiate immunogenic tumor cell death and enhance T cell–mediated immunity in mice models (63).

Notably, the magnitude of decreased TILs seemed to be numerically larger in HER2-positive breast cancer implying a potential synergistic interaction between HER2-targeted therapy and chemotherapy regarding pattern of TIL changes over time. However, the variation in treatment combinations across eligible studies and the complex interplay between immune system and tumor in HER2-positive breast cancer preclude any firm conclusion.

Second, a potential prognostic role of dynamic TILs changes in patients with TNBC was seen. In fact, increased TILs during NACT seemed to be associated with better prognosis. Although this pooled analysis is prone to confounding bias since it was based on results from univariate rather than multivariate analyses, these findings trigger some interesting hypotheses. Currently, the presence of residual disease after NACT is the only well-established approach to optimize postoperative treatment strategy in patients with TNBC. Recently, TILs have been confirmed as having a strong prognostic value in early TNBC patients treated with chemotherapy (64)but also in early TNBC patients without chemotherapy where high TILs could identify a subset of patients with an excellent prognosis able to de-escalation strategies (65). According to our findings, increased ΔTILs after NACT might serve as an additional potential biomarker for de-escalation by defining a subgroup of patients with better prognosis and should be further validated in future studies.

Our meta-analysis has several limitations that should be considered when interpreting the results. First, all studies were retrospective with limited sample size, thus influencing the quality of current evidence and the generalizability of our findings. Second, all our pooled analyses were based on study-level results rather on individual patient data. Another limitation that deserves attention is the lack of current methodological standards for post-NAC TILs enumeration in residual cancers and pCR tumor specimens which is a source of potential methodological heterogeneity among eligible studies. In addition, TILs after NACT were counted only on residual disease in some studies whereas other counted TILs even in stroma from patients with pCR. Furthermore, some studies used TMAs that only represent small portion of tissue, which might introduce bias in heterogeneous tumors. Another source of heterogeneity among eligible studies was the various therapeutic regimens used as NACT. Considering the high between-study heterogeneity, we actively chose to use random effects model for pooled analyses as an effort to reduce the impact of heterogeneity on the pooled analyses.

Despite these caveats, our meta-analysis offers some new insights on the potential role of dynamic TILs changes after NACT in breast cancer patients that might be of clinical significance upon confirmation in further studies. In summary, we found a decreased trend in TILs through all BC subtypes after neoadjuvant treatment that might be more evident in HER2-positive breast cancer. Increased TILs might be of prognostic significance in patients with TNBC and might serve as a biomarker to identify patients with better prognosis where de-escalation strategies might be applied. Overall, dynamic TILs change evaluation on hematoxylin–eosin slides might perform as a versatile and cost-effective biomarker for breast cancer patients, specifically for HER2-positive and TNBC patients. Establishing international methodological standards on how TILs should be evaluated in residual disease and in surgical specimens with pCR is essential to be able to further validate the potential role of dynamic TILs changes after neoadjuvant therapy in future studies.

Dynamic evaluation of TILs may be of particular interest in patients with early-stage TNBC who are treated with neoadjuvant chemotherapy combined with immune checkpoint inhibitors (ICIs). The response to PD(L)-1 inhibition seems independent of PD-L1 status in the neoadjuvant setting (66), while in the GeparNuevo trial, TILs at baseline were predictive for pCR in TNBC patients receiving neoadjuvant chemotherapy with or without durvalumab, with no significant interaction between TILs and treatment arms (67). Specific chemotherapeutics may induce a stronger immunogenicity (68) and additionally, immunotherapy can induce the migration of TILs from stroma to tumor nests (67). Longitudinal evaluation of TILs might stand as an easy method to help better understand the interactions between cytotoxic and ICI and guide their combination and sequence, if prognostic correlations can be demonstrated. Further evaluating the relative proportion of specific immune subpopulations as well as the spatial organization of the immune infiltrate, including tertiary lymphoid structures may also add to the information provided by TILs enumeration and could be the future focus of the research field.

## Data availability statement

The original contributions presented in the study are included in the article/**Supplementary Material**. Further inquiries can be directed to the corresponding author.

## Author contributions

Conception and design: TF, IZ, AM, YZ; Acquisition of data: IZ, ET, YZ; Data analysis: AV, IZ, ET, YZ; Writing, review, and/or revision of the manuscript: all authors; Study supervision: IZ, AV, TF. All authors contributed to the article and approved the submitted version.

## Funding

This work was supported by grants from; the Swedish Cancer Society (grant number CAN 2018/846 and Senior Clinical Investigator award CAN 2017/1043) and the Cancer Society in Stockholm (174113) to TF; by a grant from the Swedish Breast Cancer Association to TF and IZ; AM is supported by the Swedish Cancer Society (Junior Clinical Investigator award, grant number 21 0277 JCIA 01); JB’s research group receives funding from the Stockholm region, the Swedish Cancer Society, the funds at Radiumhemmet, the Swedish Research Council, the Knut and Alice Wallenberg fund.

## Acknowledgments

We would like to thank Carl Gornitzki, Emma-Lotta Säätelä and Sabina Gillsund, librarians at Karolinska Institutet University Library, for their assistance in the search design. Part of this study was presented (online publication-only) at the American Society of Clinical Oncology (ASCO) Annual Meeting 2022.

## Conflict of interest

JB receives research funding from Merck paid to Karolinska Institutet and from Amgen, Bayer, Pfizer, Roche and Sanofi-Aventis paid to Karolinska University Hospital. No personal payments. Payment from UpToDate for a chapter in breast cancer prediction paid to Asklepios Medicine HB. TF: institutional research grants from Roche and Pfizer, institutional fees from Roche, Pfizer and Astra Zeneca and personal fees from Affibody, Novartis, Pfizer, Roche, Exact Sciences, Veracyte and UpToDate. AM: consultancy to Veracyte no financial or other compensation, AV: institutional research grant from Roche.

The remaining authors declare that the research was conducted in the absence of any commercial or financial relationships that could be construed as a potential conflict of interest.

## Publisher’s note

All claims expressed in this article are solely those of the authors and do not necessarily represent those of their affiliated organizations, or those of the publisher, the editors and the reviewers. Any product that may be evaluated in this article, or claim that may be made by its manufacturer, is not guaranteed or endorsed by the publisher.

## References

[1] Sung H Ferlay J Siegel RL Laversanne M Soerjomataram I Jemal A. Global cancer statistics 2020: Globocan estimates of incidence and mortality worldwide for 36 cancers in 185 countries. CA Cancer J Clin (2021) 71(3):209–49. doi: 10.3322/caac.21660 33538338

[2] Group EBCTC. Comparisons between different polychemotherapy regimens for early breast cancer: Meta-analyses of long-term outcome among 100 000 women in 123 randomised trials. Lancet (2012) 379(9814):432–44. doi: 10.1016/S0140-6736(11)61625-5 PMC3273723 22152853

[3] Kalinsky K Barlow WE Gralow JR Meric-Bernstam F Albain KS Hayes DF. 21-gene assay to inform chemotherapy benefit in node-positive breast cancer. New Engl J Med (2021) 385(25):2336–47. doi: 10.1056/NEJMoa2108873 PMC9096864 34914339

[4] Foukakis T Lovrot J Matikas A Zerdes I Lorent J Tobin N. Immune gene expression and response to chemotherapy in advanced breast cancer. Br J Cancer (2018) 118(4):480–8. doi: 10.1038/bjc.2017.446 PMC5830596 29370583

[5] Matikas A Lövrot J Ramberg A Eriksson M Lindsten T Lekberg T. Dynamic evaluation of the immune infiltrate and immune function genes as predictive markers for neoadjuvant chemotherapy in hormone receptor positive, Her2 negative breast cancer. Oncoimmunology (2018) 7(9):e1466017. doi: 10.1080/2162402x.2018.1466017 30228933 PMC6140817

[6] Matikas A Zerdes I Lovrot J Richard F Sotiriou C Bergh J. Prognostic implications of pd-L1 expression in breast cancer: Systematic review and meta-analysis of immunohistochemistry and pooled analysis of transcriptomic data. Clin Cancer Res (2019) 25(18):5717–26. doi: 10.1158/1078-0432.Ccr-19-1131 31227501

[7] Matikas A Zerdes I Lovrot J Sifakis E Richard F Sotiriou C. Pd-1 protein and gene expression as prognostic factors in early breast cancer. ESMO Open (2020) 5(6):e001032. doi: 10.1136/esmoopen-2020-001032 33172959 PMC7656908

[8] Zerdes I Sifakis EG Matikas A Chretien S Tobin NP Hartman J. Programmed death-ligand 1 gene expression is a prognostic marker in early breast cancer and provides additional prognostic value to 21-gene and 70-gene signatures in estrogen receptor-positive disease. Mol Oncol (2020) 14(5):951–63. doi: 10.1002/1878-0261.12654 PMC7191187 32115850

[9] Denkert C Loibl S Noske A Roller M Muller BM Komor M. Tumor-associated lymphocytes as an independent predictor of response to neoadjuvant chemotherapy in breast cancer. J Clin Oncol (2010) 28(1):105–13. doi: 10.1200/JCO.2009.23.7370 19917869

[10] Adams S Gray RJ Demaria S Goldstein L Perez EA Shulman LN. Prognostic value of tumor-infiltrating lymphocytes in triple-negative breast cancers from two phase iii randomized adjuvant breast cancer trials: Ecog 2197 and ecog 1199. J Clin Oncol (2014) 32(27):2959–66. doi: 10.1200/JCO.2013.55.0491 PMC4162494 25071121

[11] Denkert C von Minckwitz G Darb-Esfahani S Lederer B Heppner BI Weber KE. Tumour-infiltrating lymphocytes and prognosis in different subtypes of breast cancer: A pooled analysis of 3771 patients treated with neoadjuvant therapy. Lancet Oncol (2018) 19(1):40–50. doi: 10.1016/s1470-2045(17)30904-x 29233559

[12] Loi S Sirtaine N Piette F Salgado R Viale G Van Eenoo F. Prognostic and predictive value of tumor-infiltrating lymphocytes in a phase iii randomized adjuvant breast cancer trial in node-positive breast cancer comparing the addition of docetaxel to doxorubicin with doxorubicin-based chemotherapy: Big 02-98. J Clin Oncol (2013) 31(7):860–7. doi: 10.1200/JCO.2011.41.0902 23341518

[13] Salgado R Denkert C Demaria S Sirtaine N Klauschen F Pruneri G. The evaluation of tumor-infiltrating lymphocytes (Tils) in breast cancer: Recommendations by an international tils working group 2014. Ann Oncol (2015) 26(2):259–71. doi: 10.1093/annonc/mdu450 PMC6267863 25214542

[14] Hendry S Salgado R Gevaert T Russell PA John T Thapa B. Assessing tumor infiltrating lymphocytes in solid tumors: A practical review for pathologists and proposal for a standardized method from the international immuno-oncology biomarkers working group: Part 2: Tils in melanoma, gastrointestinal tract carcinomas, non-small cell lung carcinoma and mesothelioma, endometrial and ovarian carcinomas, squamous cell carcinoma of the head and neck, genitourinary carcinomas, and primary brain tumors. Adv anatomic Pathol (2017) 24(6):311. 10.1097/PAP.0000000000000161 PMC5638696 28777143

[15] Board WCoTE. Who classification of breast tumours: Who classification of tumours, volume 2. World Health Organization (2019).

[16] Lusho S Durando X Bidet Y Molnar I Kossai M Bernadach M. Perception trial protocol: Comparison of predictive and prognostic capacities of neutrophil, lymphocyte, and platelet counts and tumor-infiltrating lymphocytes in triple negative breast cancer. Medicine (2020) 99(50). 10.1097/MD.0000000000023418 PMC7738079 33327268

[17] Aly I. Nct identifer 05206396. clinicaltrialsgov (2022).

[18] Altman DG McShane LM Sauerbrei W Taube SE. Reporting recommendations for tumor marker prognostic studies (Remark): Explanation and elaboration. BMC Med (2012) 10(1):1–39. 22642691 10.1186/1741-7015-10-51 PMC3362748

[19] DerSimonian R Laird N. Meta-analysis in clinical trials. Controlled Clin trials (1986) 7(3):177–88. doi: 10.1016/0197-2456(86)90046-2 3802833

[20] Tierney JF Stewart LA Ghersi D Burdett S Sydes MR. Practical methods for incorporating summary time-to-Event data into meta-analysis. Trials (2007) 8(1):1–16. doi: 10.1186/1745-6215-8-16 17555582 PMC1920534

[21] Honkoop AH. Effects of chemotherapy on pathologic and biologic characteristics of locally advanced breast cancer. (1996). 10.1093/ajcp/107.2.211 9024070

[22] Abdel-Fatah TM McArdle SE Johnson C Moseley PM Ball GR Pockley AG. Hage (Ddx43) is a biomarker for poor prognosis and a predictor of chemotherapy response in breast cancer. Br J Cancer (2014) 110(10):2450–61. doi: 10.1038/bjc.2014.168 PMC4021517 24755885

[23] Dieci MV Criscitiello C Goubar A Viale G Conte P Guarneri V. Prognostic value of tumor-infiltrating lymphocytes on residual disease after primary chemotherapy for triple-negative breast cancer: A retrospective multicenter study. Ann Oncol (2014) 25(3):611–8. doi: 10.1093/annonc/mdt556 PMC3933248 24401929

[24] Ali HR Dariush A Provenzano E Bardwell H Abraham JE Iddawela M. Computational pathology of pre-treatment biopsies identifies lymphocyte density as a predictor of response to neoadjuvant chemotherapy in breast cancer. Breast Cancer Res (2016) 18(1):21. doi: 10.1186/s13058-016-0682-8 26882907 PMC4755003

[25] Castaneda CA Mittendorf E Casavilca S Wu Y Castillo M Arboleda P. Tumor infiltrating lymphocytes in triple negative breast cancer receiving neoadjuvant chemotherapy. World J Clin Oncol (2016) 7(5):387–94. doi: 10.5306/wjco.v7.i5.387 PMC5056330 27777881

[26] Criscitiello C Bayar MA Curigliano G Symmans FW Desmedt C Bonnefoi H. A gene signature to predict high tumor-infiltrating lymphocytes after neoadjuvant chemotherapy and outcome in patients with triple-negative breast cancer. Ann Oncol (2018) 29(1):162–9. doi: 10.1093/annonc/mdx691 29077781

[27] Dieci MV Prat A Tagliafico E Pare L Ficarra G Bisagni G. Integrated evaluation of Pam50 subtypes and immune modulation of pcr in Her2-positive breast cancer patients treated with chemotherapy and Her2-targeted agents in the cherlob trial. Ann Oncol (2016) 27(10):1867–73. doi: 10.1093/annonc/mdw262 27484801

[28] Hida AI Sagara Y Yotsumoto D Kanemitsu S Kawano J Baba S. Prognostic and predictive impacts of tumor-infiltrating lymphocytes differ between triple-negative and Her2-positive breast cancers treated with standard systemic therapies. Breast Cancer Res Treat (2016) 158(1):1–9. doi: 10.1007/s10549-016-3848-2 27260189 PMC4937092

[29] Kaewkangsadan V Verma C Eremin JM Cowley G Ilyas M Eremin O. Crucial contributions by T lymphocytes (Effector, regulatory, and checkpoint inhibitor) and cytokines (Th1, Th2, and Th17) to a pathological complete response induced by neoadjuvant chemotherapy in women with breast cancer. J Immunol Res (2016) 2016:4757405. doi: 10.1155/2016/4757405 27777963 PMC5061970

[30] Park JH Kang MJ Ahn JH Kim JE Jung KH Gong G. Phase ii trial of neoadjuvant letrozole and lapatinib in Asian postmenopausal women with estrogen receptor (Er) and human epidermal growth factor receptor 2 (Her2)-positive breast cancer [Neo-all-in]: Highlighting the tils, er expressional change after neoadjuvant treatment, and fes-pet as potential significant biomarkers. Cancer Chemother Pharmacol (2016) 78(4):685–95. doi: 10.1007/s00280-016-3107-6 27491481

[31] Goto W Kashiwagi S Asano Y Takada K Takahashi K Hatano T. Predictive value of improvement in the immune tumour microenvironment in patients with breast cancer treated with neoadjuvant chemotherapy. ESMO Open (2018) 3(6):e000305. doi: 10.1136/esmoopen-2017-000305 30233820 PMC6135412

[32] Hamy AS Pierga JY Sabaila A Laas E Bonsang-Kitzis H Laurent C. Stromal lymphocyte infiltration after neoadjuvant chemotherapy is associated with aggressive residual disease and lower disease-free survival in Her2-positive breast cancer. Ann Oncol (2017) 28(9):2233–40. doi: 10.1093/annonc/mdx309 28911063

[33] Pelekanou V Carvajal-Hausdorf DE Altan M Wasserman B Carvajal-Hausdorf C Wimberly H. Effect of neoadjuvant chemotherapy on tumor-infiltrating lymphocytes and pd-L1 expression in breast cancer and its clinical significance. Breast Cancer Res (2017) 19(1):91. doi: 10.1186/s13058-017-0884-8 28784153 PMC5547502

[34] Force J Howie LJ Abbott SE Bentley R Marcom PK Kimmick G. Early stage Her2-positive breast cancers not achieving a pcr from neoadjuvant trastuzumab- or pertuzumab-based regimens have an immunosuppressive phenotype. Clin Breast Cancer (2018) 18(5):410–7. doi: 10.1016/j.clbc.2018.02.010 29615305

[35] Hwang HW Jung H Hyeon J Park YH Ahn JS Im YH. A nomogram to predict pathologic complete response (Pcr) and the value of tumor-infiltrating lymphocytes (Tils) for prediction of response to neoadjuvant chemotherapy (Nac) in breast cancer patients. Breast Cancer Res Treat (2019) 173(2):255–66. doi: 10.1007/s10549-018-4981-x 30324273

[36] Pelekanou V Barlow WE Nahleh ZA Wasserman B Lo YC von Wahlde MK. Tumor-infiltrating lymphocytes and pd-L1 expression in pre- and posttreatment breast cancers in the swog S0800 phase ii neoadjuvant chemotherapy trial. Mol Cancer Ther (2018) 17(6):1324–31. doi: 10.1158/1535-7163.MCT-17-1005 PMC6548451 29588392

[37] Watanabe T Hida AI Inoue N Imamura M Fujimoto Y Akazawa K. Abundant tumor infiltrating lymphocytes after primary systemic chemotherapy predicts poor prognosis in estrogen receptor-Positive/Her2-Negative breast cancers. Breast Cancer Res Treat (2018) 168(1):135–45. doi: 10.1007/s10549-017-4575-z 29168063

[38] Di Cosimo S Appierto V Silvestri M Pruneri G Vingiani A Perrone F. Targeted-gene sequencing to catch triple negative breast cancer heterogeneity before and after neoadjuvant chemotherapy. Cancers (Basel) (2019) 11(11). doi: 10.3390/cancers11111753 PMC6895966 31717320

[39] Hamy AS Bonsang-Kitzis H De Croze D Laas E Darrigues L Topciu L. Interaction between molecular subtypes and stromal immune infiltration before and after treatment in breast cancer patients treated with neoadjuvant chemotherapy. Clin Cancer Res (2019) 25(22):6731–41. doi: 10.1158/1078-0432.CCR-18-3017 31515462

[40] Kurozumi S Inoue K Matsumoto H Fujii T Horiguchi J Oyama T. Prognostic utility of tumor-infiltrating lymphocytes in residual tumor after neoadjuvant chemotherapy with trastuzumab for Her2-positive breast cancer. Sci Rep (2019) 9(1):1583. doi: 10.1038/s41598-018-38272-1 30733496 PMC6367461

[41] Liu J Xu Y Yu M Liu Z Xu Y Ma G. Increased stromal infiltrating lymphocytes are associated with circulating tumor cells and metastatic relapse in breast cancer patients after neoadjuvant chemotherapy. Cancer Manag Res (2019) 11:10791–800. doi: 10.2147/CMAR.S220327 PMC6939396 31920388

[42] Luen SJ Salgado R Dieci MV Vingiani A Curigliano G Gould RE. Prognostic implications of residual disease tumor-infiltrating lymphocytes and residual cancer burden in triple-negative breast cancer patients after neoadjuvant chemotherapy. Ann Oncol (2019) 30(2):236–42. doi: 10.1093/annonc/mdy547 30590484

[43] Ochi T Bianchini G Ando M Nozaki F Kobayashi D Criscitiello C. Predictive and prognostic value of stromal tumour-infiltrating lymphocytes before and after neoadjuvant therapy in triple negative and Her2-positive breast cancer. Eur J Cancer (2019) 118:41–8. doi: 10.1016/j.ejca.2019.05.014 31302586

[44] Tokes AM Rusz O Cserni G Toth E Rubovszky G Tokes T. Influence of mutagenic versus non-mutagenic pre-operative chemotherapy on the immune infiltration of residual breast cancer. Acta Oncol (2019) 58(11):1603–11. doi: 10.1080/0284186X.2019.1633015 31271119

[45] Wang Q Xiang Q Yu L Hu T Chen Y Wang J. Changes in tumor-infiltrating lymphocytes and vascular normalization in breast cancer patients after neoadjuvant chemotherapy and their correlations with dfs. Front Oncol (2019) 9:1545. doi: 10.3389/fonc.2019.01545 32039020 PMC6987397

[46] Abdelrahman AE Rashed HE MostafaToam OA Abdelhamid MI Matar I. Clinicopathological significance of the immunologic signature (Pdl1, Foxp3+ tregs, tils) in early stage triple-negative breast cancer treated with neoadjuvant chemotherapy. Ann Diagn Pathol (2021) 51:151676. doi: 10.1016/j.anndiagpath.2020.151676 33360026

[47] Axelrod ML Nixon MJ Gonzalez-Ericsson PI Bergman RE Pilkinton MA McDonnell WJ. Changes in peripheral and local tumor immunity after neoadjuvant chemotherapy reshape clinical outcomes in patients with breast cancer. Clin Cancer Res (2020) 26(21):5668–81. doi: 10.1158/1078-0432.CCR-19-3685 PMC7642197 32826327

[48] Campedel L Blanc-Durand P Bin Asker A Lehmann-Che J Cuvier C De Bazelaire C. Prognostic impact of stromal immune infiltration before and after neoadjuvant chemotherapy (Nac) in triple negative inflammatory breast cancers (Tnibc) treated with dose-dense dose-intense nac. Cancers (Basel) (2020) 12(9). doi: 10.3390/cancers12092657 PMC7565432 32957722

[49] Grandal B Evrevin C Laas E Jardin I Rozette S Laot L. Impact of brca mutation status on tumor infiltrating lymphocytes (Tils), response to treatment, and prognosis in breast cancer patients treated with neoadjuvant chemotherapy. Cancers (Basel) (2020) 12(12). doi: 10.3390/cancers12123681 PMC7764707 33302444

[50] Kim R Kawai A Wakisaka M Sawada S Shimoyama M Yasuda N. Immune correlates of the differing pathological and therapeutic effects of neoadjuvant chemotherapy in breast cancer. Eur J Surg Oncol (2020) 46(1):77–84. doi: 10.1016/j.ejso.2019.09.146 31563296

[51] Lee H Lee M Seo JH Gong G Lee HJ. Changes in tumor-infiltrating lymphocytes after neoadjuvant chemotherapy and clinical significance in triple negative breast cancer. Anticancer Res (2020) 40(4):1883–90. doi: 10.21873/anticanres.14142 32234876

[52] Park YH Lal S Lee JE Choi YL Wen J Ram S. Chemotherapy induces dynamic immune responses in breast cancers that impact treatment outcome. Nat Commun (2020) 11(1):6175. doi: 10.1038/s41467-020-19933-0 33268821 PMC7710739

[53] Sarradin V Lusque A Filleron T Dalenc F Franchet C. Immune microenvironment changes induced by neoadjuvant chemotherapy in triple-negative breast cancers: The mimosa-1 study. Breast Cancer Res (2021) 23(1):61. doi: 10.1186/s13058-021-01437-4 34039396 PMC8157437

[54] Grandal B Mangiardi-Veltin M Laas E Lae M Meseure D Bataillon G. Pd-L1 expression after neoadjuvant chemotherapy in triple-negative breast cancers is associated with aggressive residual disease, suggesting a potential for immunotherapy. Cancers (Basel) (2021) 13(4). doi: 10.3390/cancers13040746 PMC7916886 33670162

[55] Loibl S de la Pena L Nekljudova V Zardavas D Michiels S Denkert C. Neoadjuvant buparlisib plus trastuzumab and paclitaxel for women with Her2+ primary breast cancer: A randomised, double-blind, placebo-controlled phase ii trial (Neophoebe). Eur J Cancer (2017) 85:133–45. doi: 10.1016/j.ejca.2017.08.020 PMC5640494 28923573

[56] Nuciforo P Pascual T Cortes J Llombart-Cussac A Fasani R Pare L. A predictive model of pathologic response based on tumor cellularity and tumor-infiltrating lymphocytes (Celtil) in Her2-positive breast cancer treated with chemo-free dual Her2 blockade. Ann Oncol (2018) 29(1):170–7. doi: 10.1093/annonc/mdx647 29045543

[57] Eustace AJ Madden SF Fay J Collins DM Kay EW Sheehan KM. The role of infiltrating lymphocytes in the neo-adjuvant treatment of women with Her2-positive breast cancer. Breast Cancer Res Treat (2021) 187(3):635–45. doi: 10.1007/s10549-021-06244-1 PMC8197702 33983492

[58] Griguolo G Serna G Pascual T Fasani R Guardia X Chic N. Immune microenvironment characterisation and dynamics during anti-Her2-Based neoadjuvant treatment in Her2-positive breast cancer. NPJ Precis Oncol (2021) 5(1):23. doi: 10.1038/s41698-021-00163-6 33742063 PMC7979716

[59] Hurvitz SA Caswell-Jin JL McNamara KL Zoeller JJ Bean GR Dichmann R. Pathologic and molecular responses to neoadjuvant trastuzumab and/or lapatinib from a phase ii randomized trial in Her2-positive breast cancer (Trio-us B07). Nat Commun (2020) 11(1):5824. doi: 10.1038/s41467-020-19494-2 33203854 PMC7673127

[60] Loibl S Untch M Burchardi N Huober J Sinn BV Blohmer JU. A randomised phase ii study investigating durvalumab in addition to an anthracycline taxane-based neoadjuvant therapy in early triple-negative breast cancer: Clinical results and biomarker analysis of geparnuevo study. Ann Oncol (2019) 30(8):1279–88. doi: 10.1093/annonc/mdz158 31095287

[61] Zitvogel L Apetoh L Ghiringhelli F Kroemer G. Immunological aspects of cancer chemotherapy. Nat Rev Immunol (2008) 8(1):59–73. doi: 10.1038/nri2216 18097448

[62] Rojkó L Reiniger L Téglási V Fábián K Pipek O Vágvölgyi A. Chemotherapy treatment is associated with altered pd-L1 expression in lung cancer patients. J Cancer Res Clin Oncol (2018) 144(7):1219–26. doi: 10.1007/s00432-018-2642-4 PMC11813485 29675791

[63] Novosiadly R Schaer D Amaladas N Rasmussen E Lu ZH Sonyi A. Pemetrexed enhances anti-tumor efficacy of Pd1 pathway blockade by promoting intra tumor immune response via immunogenic tumor cell death and T cell intrinsic mechanisms. AACR (2018). doi: 10.1158/1538-7445.AM2018-4549

[64] Loi S Drubay D Adams S Pruneri G Francis PA Lacroix-Triki M. Tumor-infiltrating lymphocytes and prognosis: A pooled individual patient analysis of early-stage triple-negative breast cancers. J Clin Oncol (2019) 37(7):559. doi: 10.1200/JCO.18.01010 30650045 PMC7010425

[65] de Jong VM Wang Y Ter Hoeve ND Opdam M Stathonikos N Jóźwiak K. Prognostic value of stromal tumor-infiltrating lymphocytes in young, node-negative, triple-negative breast cancer patients who did not receive (Neo) adjuvant systemic therapy. J Clin oncology: Off J Am Soc Clin Oncol (2022) 2361. doi: 10.1200/JCO.21.01536 PMC9287283 35353548

[66] Schmid P Cortes J Pusztai L McArthur H Kümmel S Bergh J. Pembrolizumab for early triple-negative breast cancer. New Engl J Med (2020) 382(9):810–21. doi: 10.1056/NEJMoa1910549 32101663

[67] Karn T Denkert C Weber KE Holtrich U Hanusch C Sinn BV. Tumor mutational burden and immune infiltration as independent predictors of response to neoadjuvant immune checkpoint inhibition in early tnbc in geparnuevo. Ann Oncol (2020) 31(9):1216–22. doi: 10.1016/j.annonc.2020.05.015 32461104

[68] Denkert C von Minckwitz G Brase JC Sinn BV Gade S Kronenwett R. Tumor-infiltrating lymphocytes and response to neoadjuvant chemotherapy with or without carboplatin in human epidermal growth factor receptor 2-positive and triple-negative primary breast cancers. J Clin Oncol (2015) 33(9):983–91. doi: 10.1200/JCO.2014.58.1967 25534375

