# Supplementary material for: Expression patterns and prognostic implications of tumor-infiltrating lymphocytes dynamics in early breast cancer patients receiving neoadjuvant therapy: A systematic review and meta-analysis
Source: Front Oncol. 2022 Nov 30;12:999843. doi: 10.3389/fonc.2022.999843 (PMC9749788; doi:10.3389/fonc.2022.999843)
Supplement: Supplementary file 1 [file DataSheet_1.docx]

Supplementary Material

**Documentation of search strategies**

**University Library search consultation group**

Date: May 2020

Topic/research question: Dynamic expression of tumor-infiltrating lymphocytes (TILs) in breast cancer patients receiving neoadjuvant therapy and their prognostic and predictive implications.

Name of researcher(s): Ioannis Zerdes, Department of Oncology-Pathology

Librarian(s): Carl Gornitzki

Databases:

1. Medline (Ovid)
2. Embase (embase.com)
3. Web of Science Core Collection
4. PubMed Central (additional search in full text)

Total number of hits:

- Before deduplication: 1,662
- After deduplication: 930
- PubMed Central: 185

1. Medline

| Interface: Ovid MEDLINE(R) and Epub Ahead of Print, In-Process & Other Non-Indexed Citations and Daily  Date of Search: 15 May 2020  Number of hits: 412  Comment: In Ovid, two or more words are automatically searched as phrases; i.e. no quotation marks are needed | Field labels   - exp/ = exploded MeSH term - / = non exploded MeSH term - .ti,ab,kf. = title, abstract and author keywords - adjx = within x words, regardless of order - * = truncation of word for alternate endings |
| --- | --- |
| \| 1 \| exp Breast Neoplasms/ \| 289709 \| \| --- \| --- \| --- \| \| 2 \| ((breast or mamma or mammary) adj4 (cancer* or carcinoma* or neoplasm* or sarcoma* or tumor* or tumour*)).ti,ab,kf. \| 349245 \| \| 3 \| or/1-2 \| 407599 \| \| 4 \| Lymphocytes, Tumor-Infiltrating/ \| 6728 \| \| 5 \| exp CD3 Complex/ \| 13489 \| \| 6 \| CD4 Antigens/ \| 14278 \| \| 7 \| CD8 Antigens/ \| 8787 \| \| 8 \| (infiltrat* adj3 lymphocyt*).ti,ab,kf. \| 22873 \| \| 9 \| (cd3 or cd4 or cd8 or derived activated cell* or stils or strtil or til or tils).ti,ab,kf. \| 206226 \| \| 10 \| or/4-9 \| 228084 \| \| 11 \| Neoadjuvant Therapy/ \| 20252 \| \| 12 \| Chemotherapy, Adjuvant/ \| 40230 \| \| 13 \| ((neoadjuvant or adjuvant or preoperat* or pre-operat*) adj3 (chemo* or therap* or treat*)).ti,ab,kf. \| 116866 \| \| 14 \| or/11-13 \| 137411 \| \| 15 \| 3 and 10 and 14 \| 412 \| | |

2. Embase

| Interface: embase.com  Date of Search: 15 May 2020  Number of hits: 528  Comment: Emtree is the controlled vocabulary in Embase | Field labels   - /exp = exploded Emtree term - /de = non exploded Emtree term - ti,ab,kw = title, abstract and author keywords - NEAR/x = within x words, regardless of order - * = truncation of word for alternate endings |
| --- | --- |
| ('breast tumor'/exp OR (((breast OR mamma OR mammary) NEAR/4 (cancer* OR carcinoma* OR neoplasm* OR sarcoma* OR tumor* OR tumour*)):ti,ab,kw))  AND  ('tumor associated leukocyte'/de OR 'cd3 antigen'/de OR 'cd4 antigen'/de OR 'cd8 antigen'/de OR ((infiltrat* NEAR/3 lymphocyt*):ti,ab,kw) OR 'cd3':ti,ab,kw OR 'cd4':ti,ab,kw OR 'cd8':ti,ab,kw OR 'derived activated cell*':ti,ab,kw OR stils:ti,ab,kw OR strtil:ti,ab,kw OR til:ti,ab,kw OR tils:ti,ab,kw)  AND  ('neoadjuvant therapy'/exp OR 'adjuvant chemotherapy'/exp OR (((neoadjuvant OR adjuvant OR preoperat* OR 'pre-operat*') NEAR/3 (chemo* OR therap* OR treat*)):ti,ab,kw))  AND  ('article'/it OR 'article in press'/it OR 'review'/it) | |

3. Web of Science Core Collection

| Interface: Clarivate Analytics  Date of Search: 15 May 2020  Number of hits: 722 | Field labels   - TS/Topic = title, abstract, author keywords and Keywords Plus - NEAR/x = within x words, regardless of order - * = truncation of word for alternate endings   Note: sometimes “quotation marks” are needed for single search terms to avoid automatic term mapping (lemmatization). |
| --- | --- |
| TS=((breast OR mamma OR mammary) NEAR/4 (cancer* OR carcinoma* OR neoplasm* OR sarcoma* OR tumor* OR tumour*))  AND  TS=(infiltrat* NEAR/3 lymphocyt*) OR TS=(cd3 OR cd4 OR cd8 OR "derived activated cell*" OR stils OR strtil OR til OR tils)  AND  TS=((neoadjuvant OR adjuvant OR preoperat* OR "pre-operat*") NEAR/3 (chemo* OR therap* OR treat*))  Refined by: DOCUMENT TYPES: ( ARTICLE OR EARLY ACCESS OR REVIEW )  Indexes=SCI-EXPANDED, SSCI, A&HCI, CPCI-S, CPCI-SSH, ESCI Timespan=All years | |

4. PubMed Central

| Comment: Additional search in full text  Date of Search: 15 May 2020  Number of hits: 185 |  |
| --- | --- |
| (((((tumor infiltrating lymphocytes[Body - All Words] OR tumour infiltrating lymphocytes[Body - All Words] OR tils[Body - All Words] OR stils[Body - All Words]) NOT (tumor infiltrating lymphocytes[Title] OR tumour infiltrating lymphocytes[Title] OR tils[Title] OR stils[Title]))))  AND  ((Neoadjuvant Therapy[MeSH Terms] OR Chemotherapy, Adjuvant[MeSH Terms] OR (neoadjuvant therapy[Title] OR neoadjuvant chemotherapy[Title] OR adjuvant therapy[Title] OR adjuvant chemotherapy[Title] OR neoadjuvant therapy[Abstract] OR neoadjuvant chemotherapy[Abstract] OR adjuvant therapy[Abstract] OR adjuvant chemotherapy[Abstract])))  AND  ((breast neoplasms[MeSH Terms] OR (breast cancer[Title] OR breast neoplasms[Title] OR breast tumor*[Title] OR breast tumour*[Title] OR breast cancer[Abstract] OR breast neoplasms[Abstract] OR breast tumor*[Abstract] OR breast tumour*[Abstract])) | |

| Breast cancer subtype | N studies (n patients) | SMD | 95% CI | I^2^ |
| --- | --- | --- | --- | --- |
| Not specified | 4 (374) | 0.19 | -0.82 to 1.19 | 97 |
| Luminal | 2 (276) | NC | NC | NC |
| HER2-positive | 5 (441) | 0.32 | -0.25 to 0.88 | 92 |
| TNBC | 10 (929) | 0.25 | -0.21 to 0.71 | 90 |

SMD: standardized mean difference; CI: confidence intervals; NC: not calculated.

**Supplementary Table 1**. Pooled SMD of Tumor-infiltrating lymphocytes (TILs) pre- vs. post-treatment in matched breast cancer patients in studies presented TILs as continuous variable


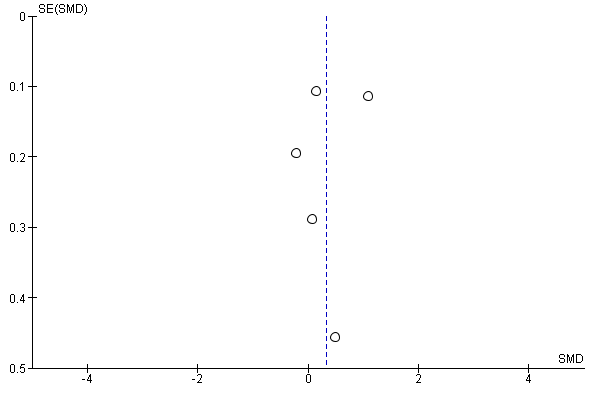
A B


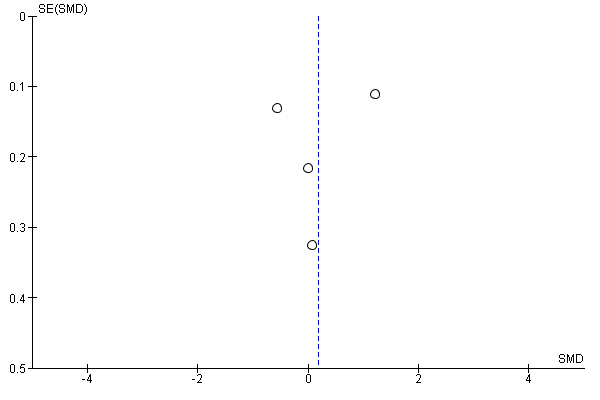


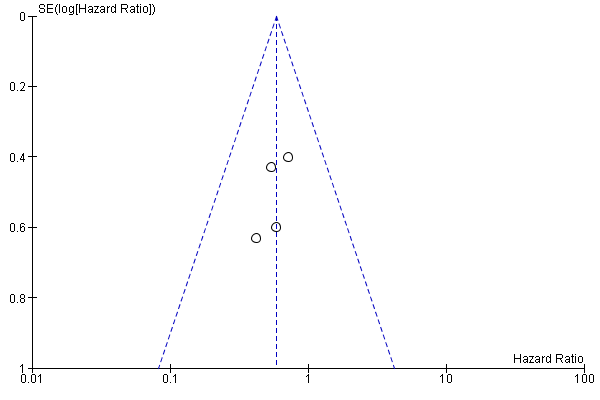
C D


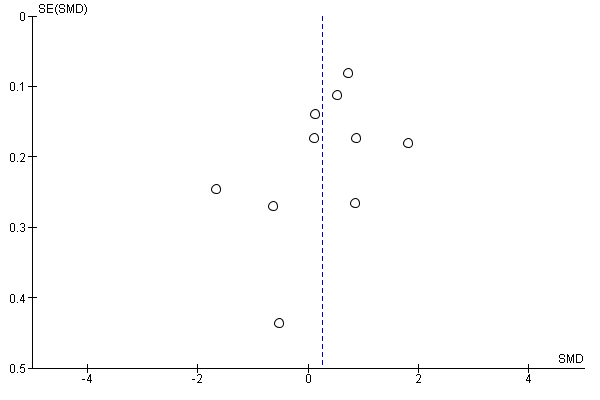


**Supplementary Figure 1**. Funnel plot analysis of potential publication bias. TILs pre- and post-treatment per in A) not specified B) HER2-positive C) Triple-negative breast cancer [TNBC] patients and D)Increased TILs vs decreased TILs for Disease-free survival [DFS]/ Recurrence-free survival [RFS] in TNBC.
